# Perceived social support and psychological wellbeing: testing the moderated mediation model of self-compassion and culture

**DOI:** 10.3389/fpsyg.2024.1474177

**Published:** 2024-12-24

**Authors:** Lubna Tannous-Haddad, Efrat Barel, Orna Tzischinsky

**Affiliations:** ^1^Behavioral Sciences Department, The Max Stern Yezreel Valley College, Yezreel Valley, Israel; ^2^School of Psychological Sciences, University of Haifa, Haifa, Israel; ^3^Educational Counseling Department, The Max Stern Yezreel Valley College (Emeritus), Yezreel Valley, Israel

**Keywords:** self-compassion, culture, wellbeing, depression, anxiety, stress

## Abstract

**Introduction:**

Self-compassion and self-coldness mediate the relationship between perceived social support and wellbeing, depression, anxiety and stress. However, this mediation has not been studied in intercultural contexts yet. The current crosssectional study explores the mediation effects of both constructs among Arabs and Jews in Israel.

**Methods:**

Participants were 496 adults—309 Jews (62.3%) and 187 Arabs (37.7%) with an average age of 40.3. Respondents completed online standardized measures of self-compassion, self-coldness, perceived social support, wellbeing, depression, anxiety, and stress. The data were analyzed using PROCESS macro model 4 and 59.

**Results:**

Testing the indirect effects of self-compassion and self-coldness on the relationship between perceived social support and wellbeing facets, we found a mediation effect for both constructs. We also found that culture moderated the mediation processes and found a stronger mediation effect for self-compassion among Arabs than Jews, but no moderation of culture on the mediation effect of self-coldness.

**Discussion:**

These results emphasize the importance of considering cultural context when designing support interventions aimed at promoting wellbeing by enhancing self-compassion. The differential mediation effect by self-compassion and self-coldness contributes to growing research pointing to a need to further explore the influence of different components of self-compassion in different cultures.

## Introduction

Perceived social support refers to individuals' subjective beliefs about the availability or adequacy of support in their social networks (Lakey and Orehek, 2011). The possible contribution of social support to wellbeing was conceptualized by Feeney and Collins (2014), whose theoretical model of thriving within relationships highlights the crucial role of relational support in both challenging and positive circumstances. The model conceptualizes social support as an interpersonal process focused on thriving, which itself is composed of hedonic, eudaimonic, psychological, social, and physical wellbeing. The model depicts two life contexts conducive to thriving: effectively coping with life's challenges and actively pursuing opportunities for personal growth and advancement. This theoretical perspective emphasizes the importance of support quality and responsiveness, assuming that individuals who receive such support are likely to thrive across various domains, including mental and physical wellbeing, relationships, and personal development.

Studies have found perceived social support to be positively linked to better psychological wellbeing (Alsubaie et al., 2019) and negatively associated with depression (Suwinyattichaiporn and Johnson, 2022), anxiety (Metts et al., 2024), and stress (Hou et al., 2023; Suwinyattichaiporn and Johnson, 2022). However, the connection between perceived social support and various aspects of wellbeing is not necessarily direct; rather, it may be mediated by such factors as self-esteem (Poudel et al., 2020), savoring (Wilson et al., 2020), and self-compassion (Chu et al., 2021; Toplu-Demirtaş et al., 2018; Wilson et al., 2020).

### Self-compassion and wellbeing

When humans face failure, adversity, or any other form of suffering, they tend to feel isolated from others, to be over-occupied with what they are dealing with, and to react with self-judgment. Conversely, self-compassion entails embracing a kind and nurturing attitude toward the self when undergoing difficult experiences. Neff (2022) defined self-compassion along a bipolar continuum, ranging from compassionate self-responding to uncompassionate self-responding or self-coldness, and delineated three components: (1) acknowledging suffering and failure as common aspects of humanity vs. feeling isolated; (2) mindfully experiencing the situation vs. overly identifying with it; and (3) responding with kindness and understanding toward oneself vs. self-criticism. In times of distress, different people display different levels of self-compassion or self-coldness (Neff, 2022).

Self-compassion transforms how individuals handle difficulties and emotions. Instead of engaging in self-criticism over mistakes, people who practice self-compassion respond to themselves with understanding—as they would to a close friend. This shift from harsh self-judgment to supportive self-talk directly lowers their risk of anxiety and depression. Their ability to handle stress improves because they start seeing personal struggles as normal human experiences rather than individual failures. This perspective helps them stay calmer under pressure and maintain healthier stress responses. Most importantly, self-compassion helps individuals deal with emotions in a balanced way. Rather than suppressing feelings or becoming overwhelmed, self-compassionate individuals learn to acknowledge emotions without judgment. They can sit with difficult feelings, understand them, and work through them productively. This creates a practical cycle of emotional stability—less self-criticism, better stress management, and more effective emotional regulation. Extensive research has established associations between self-compassion constructs and wellbeing or psychological distress (de Souza et al., 2020; Kotera et al., 2020). For example, in a study by Brenner et al. (2018), self-compassion was more strongly associated with wellbeing, while self-coldness was more strongly associated with distress. Although no direct link was found between self-compassion and distress, the former was found to play a buffering role, mitigating the relationship between self-coldness and distress. McKay and Walker (2021) reported a connection between self-compassion and wellbeing among 190 American of diverse ethnicities.

### Social support, self-compassion and wellbeing

Social support is likely to enhance self-compassion. When individuals receive consistent social support, they internalize patterns of kindness and understanding, making it easier to extend this same compassion to themselves. This reinforcement creates a positive cycle: social support provides models for self-kindness, while self-compassion enables deeper, more authentic social connections. Recent research highlights the important role of self-compassion in mediating the link between perceived social support and psychological states. Wilson et al. (2020) identified self-compassion as one of the key factors influencing perceived social support and psychological wellbeing outcomes. Similarly, Chan et al. (2023), who studied the influence of familial support on the rehabilitation of individuals with mental health issues and explored potential underlying mechanisms, found positive correlations between familial support and self-compassion. They also found correlations between self-compassion and reduced symptom severity, improved social and occupational functioning, more optimistic perceptions of recovery, and increased life satisfaction. Apparently, support from social connections can facilitate optimistic self-perceptions, potentially fostering self-kindness (Bandura, 1977; Stallman et al., 2018), especially when social support is provided by close others (Wu et al., 2024). Consequently, self-compassion could be an additional element explaining the correlation between perceived social support and overall wellbeing (Wilson et al., 2020).

### Self-compassion and wellbeing across cultures

Self-compassion has shown measurement invariance and is expected to act similarly across cultures (Tóth-Király and Neff, 2020). Yet, the relationship between self-compassion and culture remains inconclusive, as studies reveal varied results. Some findings support consistent levels of self-compassion and self-coldness across cultures (Birkett, 2014; Chio et al., 2021), while others show otherwise. As reported by Fung et al. (2021), Hong Kong college students exhibited higher levels of self-compassion than their American counterparts, but no difference was found between the two groups for self-coldness. In a comparison of German and South African employers, the former reported higher levels of self-compassion (Kotera et al., 2021). Recently in a study comparing mental health, shame and self-compassion between Indonesian and U.K. students, the former reported higher levels of self-compassion (Kotera et al., 2024).

Studies have also found varied associations between self-compassion constructs and mental health facets in different cultures. Fung et al. (2021) found self-coldness to be associated with increased depression and anxiety and decreased wellbeing among college students in both Hong Kong and the US, but the negative association between self-coldness and wellbeing was significantly larger among the former. In a study by Boyraz et al. (2020), Hispanic/Latinx and European American college students reported negative associations between self-criticism and perceived health, while Asian Americans did not. Once adjusted for self-criticism, the correlation between self-compassion and perceived health was found to be positive among both Asian Americans and European Americans, but not Hispanic/Latinx participants, and this relationship was stronger for Asian Americans. Another study found self-compassion to be more strongly associated with positive emotions (feelings of happiness, joy, and contentment) among adults in the United States than in Japan (Arimitsu et al., 2019). A descriptive study of Japanese and Dutch employees found self-compassion to negatively predict mental health problems among the former, whereas work engagement negatively predicted mental health problems among the latter (Fung et al., 2021; Kotera et al., 2021). This wide body of research endorses the role of culture as a moderator of the relationships between self-compassion, self-coldness, psychological wellbeing, and psychological distress.

Cultural differences in self-compassionate behavior can be explained by Cultural Dimensions Theory (Hofstede, 2001; Hofstede et al., 2010), which profiles cultures through six dimensions: (1) individualism–collectivism (the degree to which individuals are independent or interdependent); (2) long-term orientation (the degree to which individuals are future oriented and invest in preparing for it); (3) uncertainty avoidance (the extent of tolerance that society members have for uncertainty); (4) masculinity–femininity (the extent to which individuals prioritize tasks and competitive accomplishments as opposed to focusing on interpersonal relationships and connections with others); (5) power distance (the degree to which hierarchy is an acceptable norm); and (6) indulgence–restraint (the degree to which society embraces hedonism and impulsive behavior). The way a culture perceives each dimension can affect a person's self-compassion and self-coldness behavior. In a study in 21 countries of the relationship between Hofstede's dimensions and self-compassion, Montero-Marin et al. (2018) found higher long-term orientation and uncertainty avoidance to be associated with higher self-compassion scores, while high levels of indulgence were negatively correlated with self-compassion. According to Fung et al. (2021), people in cultures that emphasize interdependence tend to exhibit greater levels of self-compassion. This could be attributed to cultural norms valuing relationship harmony and shared humanity among individuals.

### The current study

Studies show that self-compassion might be perceived differently in different cultures (e.g., Montero-Marin et al., 2018). However, it has not been explored in the context of Jews and Arabs in Israel. Israel has a majority of Jews (78%) and a minority of Arabs (22%). Although both groups are citizens of the country, they represent two different cultures. Previous studies suggested that Arabs and Jews differ in several aspects of cultural dimensions, especially those found to be associated with self-compassion. For example, the Arab culture is collectivist the Jewish culture is individualist (Sagie et al., 2005). Arabs have been found to demonstrate higher levels of uncertainty avoidance in comparison to Jews (e.g., Abu-Saad et al., 2020; Qiadan et al., 2012). Furthermore, cultural differences in long-term orientation have been documented between Islamic vs. non-Islamic countries, with Islamic countries showing higher long-term orientation (Díez-Esteban et al., 2019). As presented earlier, higher long-term orientation, uncertainty avoidance and lower levels of indulgence were associated with higher self-compassion scores. Therefore, it is assumed that Arabs and Jews will differ in self-compassion construct.

Arabs as a minority group in Israel, have been associated with higher levels of anxiety and distress, and lower levels of resilience, when compared to Jews (Marciano et al., 2019). Research shows that perceived social support is associated with wellbeing and psychological distress and that this association is mediated by self-compassion and self-coldness (Chu et al., 2021; Wilson et al., 2020). However, according to recent findings, the associations of self-compassion and self-coldness with wellbeing facets work differently in different cultures (Arimitsu, 2023; Fung et al., 2021). To the best of our knowledge the moderated mediation of self-compassion and culture on the relationship between perceived social support and psychological wellbeing has not been studied yet. Therefore, the main goal of the current study is to examine how self-compassion and self-coldness mediate the relationship between perceived social support and wellbeing among Arabs and Jews in Israel. Our main hypotheses were:

H1: Perceived social support will correlate positively with wellbeing and negatively with depression, anxiety, and stress.
H2: Self-compassion and self-coldness will mediate the relationship between perceived social support and wellbeing, depression, anxiety, and stress.
H3: The mediation effect of self-compassion and self-coldness on the relationship between perceived social support and wellbeing, depression, anxiety, and stress will be moderated by culture.

## Method and materials

### Participants

Participants included 496 Israeli adults recruited online: 309 Jews (62.3%) and 187 Arabs (37.7%), of whom 49% were male. To be included, participants had to be 20–60 years old and speak the language in which the survey was administered (Hebrew or Arabic). No exclusion criteria were applied. The mean age of respondents was 40.42 (SD = 12.01). The mean of years of education was 13.78 (SD = 3.09).

### Procedure

The authors collected the data via in a cross-sectional survey conducted in Israel, in July 2024. Qualtrics (https://www.qualtrics.com) was used to create an anonymous questionnaire, which was distributed online by iPanel (https://www.ipanel.co.il), a large Israeli panel service, an online research platform with access to a probability-based panel of ~100,000 Israeli participants, enabling representative sampling of Israeli society. To ensure demographic balance, quotas were established prior to data collection based on key variables from Israel's Central Bureau of Statistics (CBS) census data, including sex and ethnicity. The survey remained open until both the required sample size and predetermined demographic quotas were met, ensuring the sample's distribution matched national demographics. Participants received monetary compensation for their involvement through the platform's standard protocol. The complete study protocol was approved by the College Institutional Review Board. Questionnaire completion was voluntary, and respondents were told they could stop their participation at any point. Data from 24 participants who completed the survey were excluded from the final analysis if their responses were implausible (e.g., they chose the same answer throughout the questionnaire) or incomplete. The final analysis included 496 participants.

### Measures

#### Demographics

The demographic questionnaire included items on culture (Jew or Arab), parenthood, gender, age, residence, religion, and education.

#### Perceived social support

Perceived social support was measured by the *Multidimensional Scale of Perceived Social Support* (*MSPSS*; Zimet et al., 1988; α=0.94). This 12-item instrument is comprised of three subscales that measure support from family (e.g., “My family really tries to help me”), friends (e.g., “I can count on my friends when things go wrong”), and significant others (e.g., “I have a special person who is a real source of help for me”). Responses fall along a scale of 1 (strongly disagree) to 7 (strongly agree). The mean score is computed such that a higher score reflects greater support. The MSPSS was translated to Hebrew and adapted to Israel by Statman (1995), who reported high internal reliability (0.93 for the family subscale, 0.91 for the friends' subscale and 0.91 for the significant others subscale). Internal reliability (Cronbach's alpha) in the present study was 0.95 For the current study, the scale was translated, in two parallel processes, from English to Arabic and back, by two professionals fluent in the two languages (Cha et al., 2007).

#### Self-compassion and self-coldness

Self-compassion and self-coldness were assessed by the *Self-Compassion Scale–Short Form* (Raes et al., 2011). This 12-item questionnaire is comprised of 6 two-item subscales. The component of self-compassion is measured by the subscales of self-kindness (e.g., “I try to be understanding and patient toward those aspects of my personality I don't like”), common humanity (e.g., “I try to see my failings as part of the human condition”), and mindfulness (e.g., “When something painful happens I try to take a balanced view of the situation”). The component of self-coldness is measured by the subscales of self-judgment (e.g., “I'm intolerant and impatient toward those aspects of my personality I don't like”), isolation (e.g., “ When I'm feeling down, I tend to feel like most other people are probably happier than I am”), and over-identification (e.g., “When I'm feeling down, I tend to obsess and fixate on everything”). Responses fall along a scale of 1 (almost never) to 5 (almost always). To compute a composite score, the negative items were reversed scored, and the sum across all items was computed. Higher scores indicate greater self-compassion. Internal reliability (Cronbach's alpha) in the present study was 0.78. For the current study, the scale was translated, in two parallel processes, from English to Hebrew and back, and from Hebrew to Arabic and back, by two professionals fluent in the three languages (Cha et al., 2007).

#### Wellbeing

Wellbeing was measured by the *Mental Health Continuum–Short Form* (*MHC-SF*; Lamers et al., 2011), which assesses positive mental health over the past month. This instrument includes three dimensions: emotional wellbeing (e.g., “Satisfied with life”), social wellbeing (e.g., “That you had something important to contribute to society”), and psychological wellbeing (e.g., “That you liked most parts of your personality”). All 14 items are based on the question: “During the past month, how often did you feel …?” Possible responses fall on a 5-point Likert scale, ranging from never (0) to everyday (5). A total score was calculated for all items, with a possible range of 0–70, where a higher score indicates a higher level of positive mental health. Internal reliability (Cronbach's alpha) in the current study was 0.91. The Hebrew version was translated from English by Shrira et al. (2016). The Arabic version of the MHC-SF was retrieved from the Israeli Ministry of Health website (https://www.gov.il/BlobFolder/dynamiccollectorresultitem/psyc-ogdan/he/subjects_medical-professions-licensing_forms_ogdan.pdf).

#### Depression, anxiety, and stress

Depression, anxiety, and stress were assessed by the *Depression, Anxiety, and Stress Scale*−*21 Items* (*DASS-21*; Lovibond and Lovibond, 1995). In the current study we used a Hebrew and Arabic versions, retrieved from the DASS21 website (http://www2.psy.unsw.edu.au/dass/). Referring to the past week, the 21 statements evaluate depression (7 items; e.g., “I felt sad and depressed”), anxiety (7 items; e.g., “I was aware of dryness of my mouth”), and stress (7 items; e.g., “I tended to over-react to situations”). Responses fall along a 4-point Likert scale, ranging from never (0) to most all the time (3). A score above 11 on the depression scale indicates severe depression; a score above 8 on the anxiety scale indicates severe anxiety; and a score above 9 on the stress scale indicates moderate to severe stress. Internal reliability (Cronbach's alpha) in this study was 0.91 for depression, 0.91 for anxiety, and 0.91 for stress.

### Data analysis

#### Testing for mediation effects

Model 4 of the PROCESS macro (Hayes and Scharkow, 2013) was used to test indirect effects of self-compassion and self-coldness in the relationship between perceived social support and wellbeing dimensions. We used 95% confidence intervals with 5,000 bootstrap samples.

#### Testing for moderated mediation models

To test the assumption that the mediated effects of self-compassion and self-coldness on perceived social support and aspects of wellbeing are moderated by culture, we estimated parameters for three regression models, using model 59 of the PROCESS macro (Hayes and Scharkow, 2013), for each dependent variable: wellbeing, depression, anxiety, and stress. Given that age and years of education were associated with several key variables, they were included as covariates in the analyses. We used 95% confidence intervals with 5,000 bootstrap samples. Specifically, the regressions estimated the moderating effect of culture on the relation between perceived social support and each dependent variable (model 1); the relation between perceived social support and self-compassion/self-coldness (model 2); and the relation between self-compassion/self-coldness and each dependent variable (model 3).

## Results

Independent *t*-tests for culture differences in study variables revealed significant differences between Jews and Arabs in the level of perceived social support, self-compassion (as a subscale), anxiety, and depression. Jews reported higher levels of perceived social support, while Arabs reported higher levels of self-compassion, anxiety, and depression. No differences were found for self-coldness, wellbeing, or stress. Means, standard deviations, and bivariate associations between all key variables are presented in Table 1.

**Table 1:** Means (*M*), standard deviations (*SD*), and zero-order correlations among variables.

								**Jews**		**Arabs**		
	**1**	**2**	**3**	**4**	**5**	**6**	**7**	***M***	***SD***	***M***	***SD***	***t***
1 Perceived social support		0.34^***^	−0.23^***^	0.49^***^	−0.37^***^	−0.29^***^	−0.25^***^	5.38	1.41	4.89	1.45	3.70^***^
2 Self-compassion	0.52^***^		−0.10	0.46^***^	−0.25^***^	−0.10	−0.19^***^	3.06	0.73	3.21	0.87	2.00^*^
3 Self-coldness	−0.20^**^	−0.10		−0.35^***^	0.65^***^	0.50^***^	0.63^***^	2.64	0.80	2.58	0.83	0.87
4 Wellbeing	0.60^***^	0.58^***^	−0.28^***^		−0.50^***^	−0.32^***^	−0.39^***^	3.84	1.09	3.78	1.07	0.59
5 Depression	−0.33^***^	−0.36^***^	0.56^***^	−0.43^***^		0.89^***^	0.80^***^	11.66	4.99	12.71	5.35	2.15^*^
6 Anxiety	−0.29^***^	−0.34^***^	0.50^***^	−0.37^***^	0.76^***^		0.71^***^	8.78	3.62	10.73	4.52	5.03^***^
7 Stress	−0.27^***^	−0.31^***^	0.58^***^	−0.36^***^	0.87^***^	0.84^***^		12.94	4.88	13.71	5.18	1.64

Correlations for the Jewish sample appear above the diagonal; correlations for the Arab sample appear below the diagonal.

^*^p < 0.05, ^**^p < 0.01, ^***^p < 0.001.

### Mediation effects

Table 2 shows the results of the mediation effect tests. After controlling for age and education level, perceived social support positively predicted self-compassion and negatively predicted self-coldness. Furthermore, perceived social support positively predicted psychological wellbeing and negatively predicted depression, anxiety, and stress. Self-compassion, in turn, positively predicted wellbeing and negatively predicted depression and stress, while self-coldness negatively predicted wellbeing and positively predicted depression, anxiety, and stress.

**Table 2:** The mediation effects of perceived social support on wellbeing, depression, anxiety, and stress.

	**Self-compassion (M1)**			**Self-coldness (M2)**			**Wellbeing (Y)**		
**Predictors**	**B**	**SE**	***p***	**B**	**SE**	***p***	**B**	**SE**	***p***
Perceived social support (X)	0.21	0.02	0.000	−0.14	0.02	0.000	0.26	0.03	0.000
Self-compassion (M1)							0.46	0.05	0.000
Self-coldness (M2)							−0.26	0.05	0.000
	*R^2^ =* 0.16	*F =* 29.90	*p =* 0.000	*R^2^ =* 0.08	*F =* 14.36	*p =* 0.000	*R^2^ =* 0.43	*F =* 71.80	*p =* 0.000
	**Self-compassion (M1)**			**Self-coldness (M2)**			**Depression (Y)**		
Perceived social support (X)	0.21	0.02	0.000	−0.14	0.02	0.000	−0.03	0.00	0.000
Self-compassion (M1)							−0.03	0.01	0.000
Self-coldness (M2)							0.11	0.01	0.000
	*R^2^ =* 0.16	*F =* 29.90	*p =* 0.000	*R^2^ =* 0.08	*F =* 14.36	*p* = 0.000	*R^2^ =* 0.46	*F =* 83.13	*p =* 0.000
	**Self-compassion (M1)**			**Self-coldness (M2)**			**Anxiety (Y)**		
Perceived social support (X)	0.21	0.02	0.000	−0.14	0.02	0.000	−0.03	0.01	0.000
Self-compassion (M1)							−0.01	0.01	0.321
Self-coldness (M2)							0.08	0.01	0.000
	*R^2^ =* 0.16	*F =* 29.90	*p =* 0.000	*R^2^ =* 0.08	*F =* 14.36	*p =* 0.000	*R^2^ =* 0.30	*F =* 40.82	*p =* 0.000
	**Self-compassion (M1)**			**Self-coldness (M2)**			**Stress (Y)**		
Perceived social support (X)	0.21	0.02	0.000	−0.14	0.02	0.000	−0.01	0.00	0.002
Self-compassion (M1)							−0.03	0.01	0.001
Self-coldness (M2)							0.11	0.01	0.000
	*R^2^ =* 0.16	*F =* 29.90	*p =* 0.000	*R^2^ =* 0.08	*F =* 14.36	*p =* 0.000	*R^2^ =* 0.41	*F =* 67.18	*p =* 0.000

X, predictor; M1, mediator 1; M2, mediator 2.

The bias-corrected percentile bootstrap method suggested that the indirect effect of perceived social support on wellbeing dimensions via self-compassion was statistically significant (wellbeing: B = 0.10 (0.02), 95% CI [0.07, 0.13]; depression: B = −0.01 (0.00), 95% CI [−0.01, −0.00]; stress B = −0.05 (0.02), 95% CI [−0.08, −0.02]), with the exception of anxiety (B = −0.00 (0.00), 95% CI [−0.01, 0.00]). That is, the higher the level of perceived social support, the higher the level of self-compassion, which in turn was related to higher levels of wellbeing and lower levels of depression and stress. In addition, the indirect effect of perceived social support on wellbeing dimensions via self–coldness was statistically significant for all variables (wellbeing: B = 0.04 (0.01), 95% CI [0.02, 0.07]; depression: B = −0.02 (0.00), 95% CI [−0.02, −0.01]; anxiety: B = −0.01 (0.00), 95% CI [−0.02, −0.01]; stress: B = −0.14 (0.03), 95% CI [−0.20, −0.08]). That is, the higher the level of perceived social support, the lower the level of self-coldness, which in turn was related to higher levels of wellbeing and lower levels of depression, anxiety, and stress.

### Moderated mediation effects of culture

Table 3 provides estimates of the moderating effect of culture on: the relation between perceived social support and each dependent variable (model 1); the relation between perceived social support and self-compassion/self-coldness (model 2); and the relation between self-compassion/self-coldness and each dependent variable (model 3). As age and years of education were associated with several key variables, they were included as covariates in the analyses. In addition, Table 4 displays the index of moderated mediation values.

**Table 3:** Indirect effects of perceived social support on wellbeing, depression, anxiety, and stress through self-compassion and self-coldness.

	**Self-compassion (M1)**			**Self-coldness (M2)**			**Wellbeing (Y)**		
**Predictors**	**B**	**SE**	***p***	**B**	**SE**	***p***	**B**	**SE**	***p***
Perceived social support (X)	0.32	0.04	0.000	−0.15	0.04	0.000	0.28	0.05	0.000
Self-compassion (M1)							0.45	0.08	0.000
Self-coldness (M2)							−0.18	0.08	0.020
Culture (W)	−0.27	0.07	0.000	0.15	0.07	0.042	0.01	0.08	0.884
X × W	−0.16	0.05	0.001	−0.00	0.05	0.960	−0.02	0.06	0.710
M1 × W							−0.02	0.11	0.883
M2 × W							−0.14	0.10	0.160
	*R^2^ =* 0.20	*F =* 24.06	*p =* 0.000	*R^2^ =* 0.09	*F =* 9.50	*p =* 0.000	*R^2^ =* 0.43	*F =* 39.96	*p =* 0.000
	**Self-compassion (M1)**			**Self-coldness (M2)**			**Depression (Y)**		
Perceived social support (X)	0.32	0.04	0.000	−0.15	0.04	0.000	−0.02	0.01	0.012
Self-compassion (M1)							−0.05	0.01	0.000
Self-coldness (M2)							0.11	0.01	0.000
Culture (W)	−0.27	0.07	0.000	0.15	0.07	0.042	−0.04	0.01	0.004
X × W	−0.16	0.05	0.001	−0.00	0.05	0.960	−0.01	0.01	0.579
M1 × W							0.02	0.02	0.191
M2 × W							0.01	0.02	0.381
	*R^2^ =* 0.20	*F =* 24.06	*p =* 0.000	*R^2^ =* 0.09	*F =* 9.50	*p =* 0.000	*R^2^ =* 0.47	*F =* 47.97	*p =* 0.000
	**Self-compassion (M1)**			**Self-coldness (M2)**			**Anxiety (Y)**		
Perceived social support (X)	0.32	0.04	0.000	−0.15	0.04	0.000	−0.02	0.01	0.035
Self-compassion (M1)							−0.04	0.01	0.001
Self-coldness (M2)							0.09	0.01	0.000
Culture (W)	−0.27	0.07	0.000	0.15	0.07	0.042	−0.08	0.01	0.000
X × W	−0.16	0.05	0.001	−0.00	0.05	0.960	−0.00	0.01	0.842
M1 × W							0.05	0.02	0.007
M2 × W							−0.01	0.02	0.577
	*R^2^ =* 0.20	*F =* 24.06	*p =* 0.000	*R^2^ =* 0.09	*F =* 9.50	*p =* 0.000	*R^2^ =* 0.36	*F =* 24.57	*p =* 0.000
	**Self-compassion (M1)**			**Self-coldness (M2)**			**Stress (Y)**		
Perceived social support (X)	0.32	0.04	0.000	−0.15	0.04	0.000	−0.01	0.01	0.131
Self-compassion (M1)							−0.04	0.01	0.002
Self-coldness (M2)							0.10	0.01	0.000
Culture (W)	−0.27	0.07	0.000	0.15	0.07	0.042	−0.03	0.01	0.019
X × W	−0.16	0.05	0.001	−0.00	0.05	0.960	0.00	0.01	0.880
M1 × W							0.02	0.02	0.338
M2 × W							0.01	0.02	0.435
	*R^2^ =* 0.20	*F =* 24.06	*p =* 0.000	*R^2^ =* 0.09	*F =* 9.50	*p =* 0.000	*R^2^ =* 0.42	*F =* 38.41	*p =* 0.000

X, predictor; M1, mediator 1; M2, mediator 2; W, moderator.

**Table 4:** Index of the moderated mediation of perceived social support on wellbeing, depression, anxiety, and stress for different levels of cultural group.

	**Index**	**Boot SE**	**Boot LLCI**	**Boot ULCI**
**Wellbeing**				
Self-compassion	−0.07	0.04	−0.15	−0.00
Self-coldness	0.02	0.03	−0.03	0.07
**Depression**				
Self-compassion	0.01	0.01	0.00	0.02
Self-coldness	0.00	0.01	−0.11	0.13
**Anxiety**				
Self-compassion	0.01	0.01	0.00	0.03
Self-coldness	0.00	0.01	−0.11	0.13
**Stress**				
Self-compassion	0.01	0.01	−0.00	0.02
Self-coldness	−0.00	0.01	−0.02	0.01

LLCI, lower limit confidence interval; ULCI, upper limit confidence interval.

#### Wellbeing

**Figure 1:**
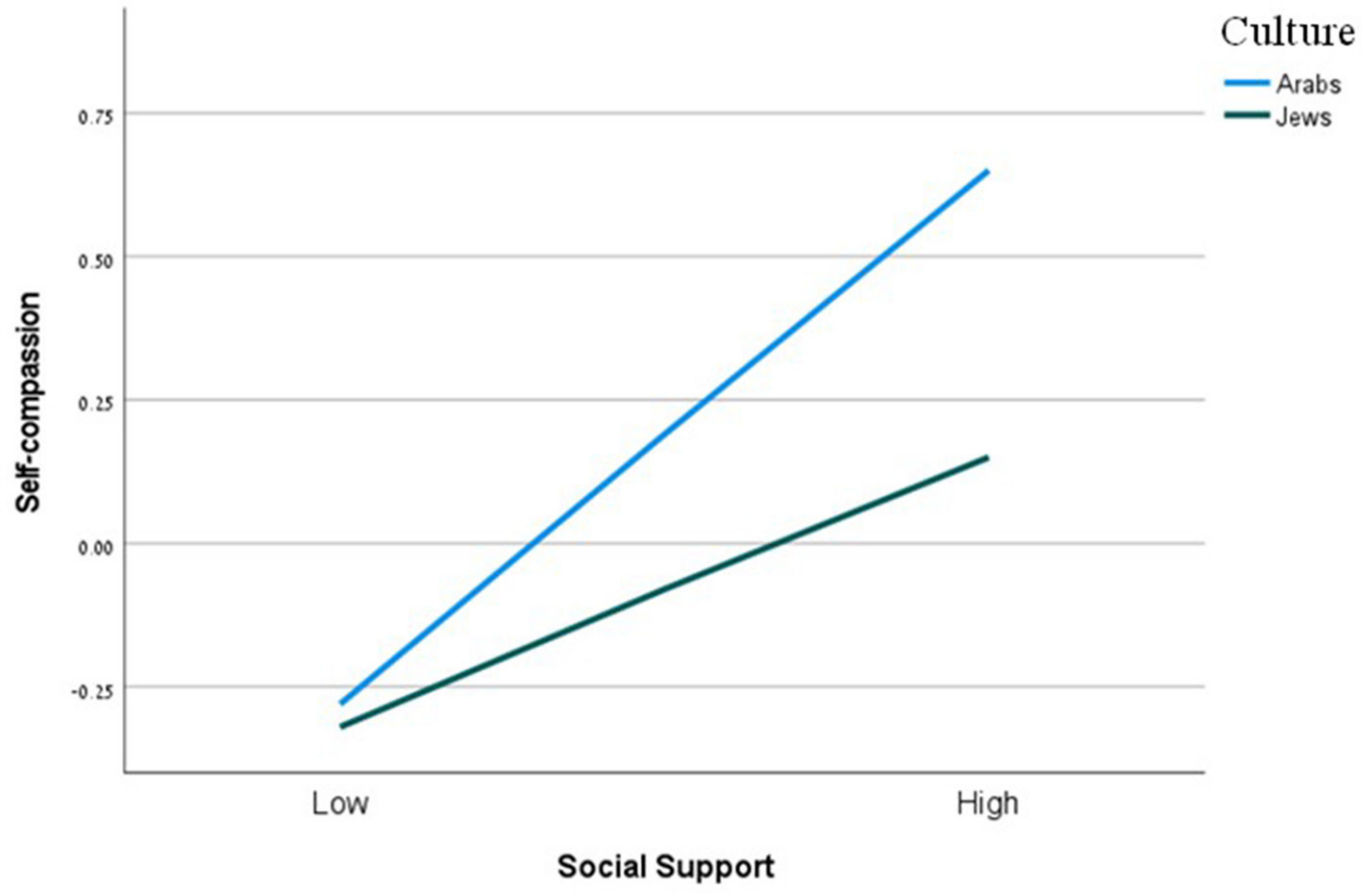
The moderating effect of culture on the relation between perceived social support and self-compassion. Functions are graphed for two levels of culture: Arabs and Jews.

As displayed in Table 3, there was a significant main effect of perceived social support on wellbeing, and this effect was not moderated by culture. Looking at the effect of perceived social support on each of the mediators, we found, firstly, a significant effect on self-compassion, moderated by culture. Simple slope tests showed the association between perceived social support and self-compassion to be stronger for Arabs (*b* =0.32, se =0.04, 95/% CI = [0.25,0.39]) than Jews (*b* = 0.16, se = 0.03, 95/% CI = [0.10,0.22]; see Figure 1). The effect of perceived social support on self-coldness was also significant, but this effect was not moderated by culture. Next, we examined the moderating effect of culture on the relation between each mediator and wellbeing. The interactions between self-compassion and culture, and between self-coldness and culture, on wellbeing were nonsignificant. The index of moderated mediation was only significant for self-compassion, meaning that the indirect effect is conditional on the level of cultural group only for self-compassion (see Table 4).

#### Depression

As Table 3 shows, there was also a significant main effect of perceived social support on depression. Like the case of wellbeing, this effect was not moderated by culture. With respect to the effect of such support on each of the mediators, this effect was significant for self-compassion, moderated by culture. Simple slope tests showed the association between perceived social support and self-compassion to be stronger for Arabs than Jews (Table 3). The effect of perceived social support on self-coldness was also significant, but not moderated by culture. Looking at the moderating effect of culture on the relation between each mediator and depression, we found nonsignificant interactions between self-compassion and culture, and between self-coldness and culture, on depression. As with wellbeing, the index of moderated mediation was only significant for self-compassion (Table 4).

#### Anxiety

**Figure 2:**
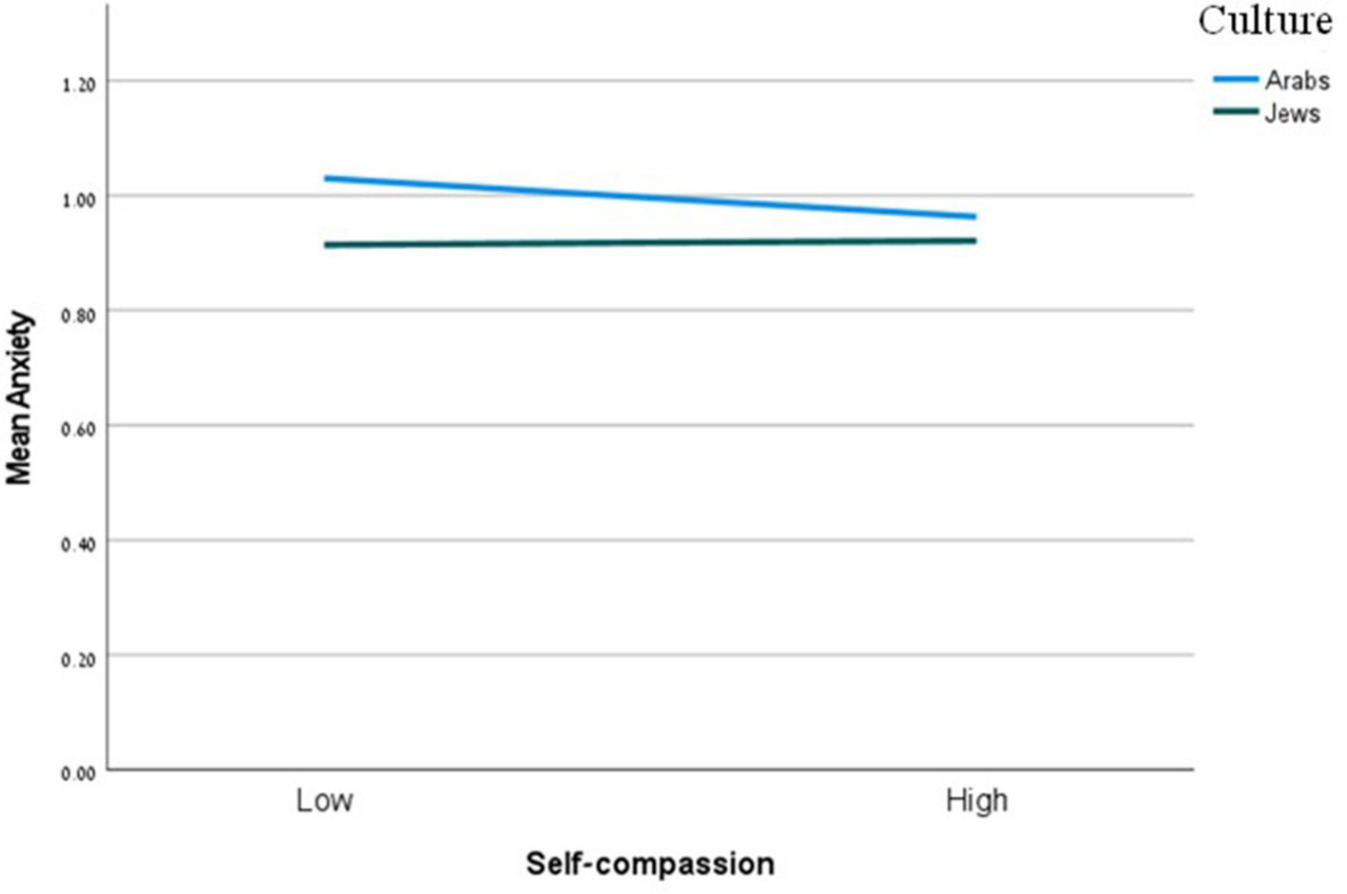
The moderating effect of culture on the relation between self-compassion and anxiety. Functions are graphed for two levels of culture: Arabs and Jews.

Table 3 also reveals a significant main effect of perceived social support on anxiety, and, again, this effect was not moderated by culture. The effect of perceived social support on self-compassion was significant and moderated by culture. Simple slope tests showed the association between such support and self-compassion to be stronger for Arabs than Jews (Figure 1). The effect of perceived social support on self-coldness was significant, but this effect was not moderated by culture. The interaction between self-compassion and culture on anxiety was significant. Simple slope tests showed that the association between self-compassion and anxiety was significant only for Arabs (*b* = −0.04, se =0.01, 95/% CI = [−0.07, −0.02]), and not for Jews (*b* = 0.00, se = 0.01, 95/% CI = [−0.02, 0.23]). That is, for Arabs, the higher the self-compassion levels, the lower the anxiety levels (Figure 2). The interaction between self-coldness and culture on anxiety was nonsignificant. Again, the index of moderated mediation was only significant for self-compassion (Table 4).

#### Stress

As shown in Table 3, the effect of perceived social support on stress was not significant and was not moderated by culture. The effect of perceived social support on self-compassion was significant, and this effect was moderated by culture. Simple slope tests showed the association between perceived social support and self-compassion was stronger for Arabs that for Jews (see Table 3). The effect of perceived social support on self-coldness was significant, but this effect was not moderated by culture. The interactions between self-compassion and culture, and between self-coldness and culture, on stress were nonsignificant. Finally, the index of moderated mediation was nonsignificant for both self-compassion and self-coldness (Table 4).

## Discussion

The aim of this study was to examine how self-compassion and self-coldness mediate the relationship between perceived social support and wellbeing among Arabs and Jews in Israel. First, findings indicated some cultural differences: Arabs reported higher levels of self-compassion, anxiety, and depression, while Jews reported higher levels of perceived social support. The high levels of anxiety and depression among Arabs are consistent with previous research and can be related to their status as a minority group in Israel (Kaplan et al., 2019; Tannous-Haddad et al., 2022). Their lower level of perceived social support can be attributed to the interconnectedness in collectivist societies, which might create high expectations of others, leading in turn to a sense of less support. The intercultural differences in terms of reported self-compassion are in line with previous studies (Birkett, 2014; Chio et al., 2021). In keeping with Hofstede's Cultural Dimensions Theory (Hofstede, 2001; Hofstede et al., 2010), studies have associated high levels of self-compassion with collective cultures with high uncertainty avoidance (Arimitsu, 2023; Montero-Marin et al., 2018). Indeed, this may describe the Arab culture in Israel, whereas the Jewish culture is characterized by individualism and high low uncertainty avoidance. Interestingly, the current study found no cultural differences in self-coldness. Similar results were reported by Fung et al. (2021). Self-compassion was linked to significantly higher levels of subjective well-being and lower levels of depressive and anxiety symptoms in Hong Kong. However, these associations were not significant among students in the United States. Conversely, self-coldness was negatively related to subjective wellbeing and positively related to depressive and anxiety symptoms in both groups. In addition, wellbeing and stress showed no cultural differences. Such results must be approached with caution as psychosocial demographic changes might be taking place (Ali, 2019).

According to Feeney and Collins (2014), social support is a major factor contributing to thriving, the higher the support, the better the individual's wellbeing. Our first hypothesis, which assumed that perceived social support would correlate positively with wellbeing and negatively with depression, anxiety, and stress, was confirmed for the most part. Our results replicated the long-known impact of perceived social support on wellbeing, depression, and anxiety (Alsubaie et al., 2019; Metts et al., 2024). The impact of perceived social support on stress was not found to be significant in this study. While much previous research has established a connection between perceived social support and stress (Hou et al., 2023; Suwinyattichaiporn and Johnson, 2022), some studies suggest that this relationship might be influenced by other factors. One such factor is negative affect. Specifically, as negative affect increases, the positive effects of social support on perceived stress tend to decrease (Çivitci, 2015).

Research has also shown that the association between social support and facets of wellbeing is mediated by self-compassion and self-coldness (Chan et al., 2023; Wilson et al., 2020). Our second hypothesis expected these constructs to mediate the relationship between perceived social support and wellbeing, depression, anxiety, and stress among Arabs and Jews alike. Results showed that self-compassion and self-coldness indeed mediated these relationships. A higher level of perceived social support was associated with a higher level of self-compassion, which in turn was related to a higher level of wellbeing and lower levels of depression and stress. A higher level of perceived social support was also associated with a lower level of self-coldness, which in turn was related to a higher level of wellbeing and lower levels of depression, anxiety, and stress. This empirical evidence reveals the complex interplay between social support, self-compassion and self-coldness, and psychological outcomes. When individuals perceive strong social support in their lives, it manifests in two distinct but parallel pathways of self-relation. First, it enhances self-compassion—individuals who feel supported by others are more likely to develop a kind, understanding relationship with themselves. This heightened self-compassion then serves as a psychological buffer, promoting overall wellbeing while reducing depressive symptoms and stress levels. The second pathway operates through the reduction of self-coldness. Strong social support appears to protect individuals from developing a harsh, critical relationship with themselves. When people experience consistent support from others, they're less likely to engage in self-judgment and isolation. This decreased self-coldness has wide-ranging benefits, contributing not only to enhanced wellbeing and reduced depression and stress (as with self-compassion), but also specifically helping to lower anxiety levels. These findings make theoretical sense: when individuals are consistently exposed to supportive others who model understanding, acceptance, and warmth, they learn to relate to themselves in similar ways. This creates a positive psychological cascade – supportive relationships foster healthier self-relating patterns, which in turn promote better mental health outcomes. The distinction between promoting self-compassion and reducing self-coldness suggests that social support works both by building positive self-relating resources and by protecting against negative self-relating patterns. Previous studies also reported that self-compassion and self-coldness mediated the relationship between perceived social support and wellbeing, anxiety, stress and depression (Chu et al., 2021; Toplu-Demirtaş et al., 2018; Wilson et al., 2020). In keeping with previous findings (Wilson et al., 2020) and with the model of Feeney and Collins (2014), our results highlight the role that self-compassion can play in enhancing the contribution of social support to wellbeing.

The third hypothesis claimed that culture would moderate the association between perceived social support and wellbeing facets through self-compassion and self-coldness. The significant main effect of perceived social support on wellbeing, depression, and anxiety was not moderated by culture, possibly because of the strong effect of social support on wellbeing, which appears to go beyond cultural differences. However, culture was found to moderate the path between perceived social support and self-compassion (but not self-coldness): there was a stronger connection between perceived social support and self-compassion for Arabs than Jews. Viewed through the lens of the Cultural Dimensions Theory, Arab culture is collectivist such that individuals are interconnected with their family, community, and society. Hence, the self and one's behavior are highly influenced by the reactions of close others to what one is undergoing (Wu et al., 2024). Individuals in such a culture aspire to a harmony between themselves and their social environment and tend to adopt collective social messages and expectations (Dwairy, 1998; Haj-Yahia, 2019). Although in general, humans tend to be non-compassionate to themselves, in collectivist societies, perceived social support seems to endorse self-compassion. A message of support legitimizes the individual's experience and promotes self-caring and belonging. At the same time, Jews belong to an individualist culture (Sagie et al., 2005) where the self is much less influenced by messages from the social environment; thus, the effect of higher perceived social support on self-compassion for Jews is less compared to Arabs.

The significant mediated associations between perceived social support and self-coldness were not moderated by culture for any of the four facets of wellbeing. This lack of cultural moderation indicates that the association between perceived social support and self-coldness acts similarly in both cultures. This lack of moderation also supports previous results of Fung et al. (2021). The varying outcomes across different cultural contexts indicate that self-compassion should be viewed as a dual-component construct. Both self-compassion and self-coldness play unique roles in influencing the psychological wellbeing of young adults from diverse cultural backgrounds.

Culture moderated the associations between perceived social support and self-compassion but not the associations between self-compassion and wellbeing facets. Nonetheless, culture should still be considered a moderator, as taking it into consideration reveals that perceived social support plays a more significant role among Arabs when self-compassion is taken into account. These findings support the changing moderating role that culture plays in the mediation process between perceived social support and wellbeing facets (Arimitsu, 2023; Kotera et al., 2020).

When exploring interactions, we only found a significant interaction between self-compassion and culture in the case of anxiety as similarly reported by Chi et al. (2023). Moreover, the association between self-compassion and anxiety was significant only for Arabs. In other words, self-compassion was found to be important for reducing anxiety only in the case of a collectivist culture. The relationship between self-compassion and anxiety appears to be culturally dependent. In collectivist cultures, where high uncertainty avoidance is prevalent, self-compassion emerges as a crucial protective factor against anxiety (Carleton, 2016). The heightened concern about uncertainty in these societies tends to intensify anxiety levels, making self-compassion's calming and accepting qualities particularly valuable. In contrast, individualistic societies, such as Jewish communities studied here, demonstrate a different pattern. These societies typically exhibit lower levels of uncertainty avoidance and consequently report lower baseline levels of anxiety. In this cultural context, the relationship between self-compassion and anxiety becomes less pronounced, suggesting that self-compassion's psychological benefits may manifest differently in societies where uncertainty is approached with less apprehension. This finding challenges assumptions about universal patterns in self-compassion's protective effects and highlights the importance of considering cultural context when understanding psychological coping mechanisms. Given that Arabs reported higher anxiety levels than Jews, this can be an excellent opening for an intervention. Teaching and empowering individuals to enhance their self-compassion can help reduce their levels of anxiety.

The current study has several limitations. For one thing, our data is based on self-reports at a single point of time. Participants may have reported while affected by a certain life event or circumstance. Moreover, the study utilized a cross-sectional design, which did not allow us to determine causal associations. We recommend undertaking longitudinal studies with several sampling points to further deepen understanding of the relationship between perceived social support and wellbeing facets, as well as the mediating role of self-compassion in cultural contexts. Furthermore, the selected sampling approach, while widely used, is biased toward individuals with a higher level of technological proficiency and accessibility to online surveys. It is essential to consider this factor when analyzing results. Another limitation is the lack of consideration of diversity within the studied groups. Both the Arab minority and Jewish majority in Israel encompass individuals from diverse subgroups, including various racial or ethnic backgrounds, religious affiliations, occupations, and social classes. Although it is accustomed within self-compassion research to study cross-cultural differences based on cultural affiliation, there is a need for exploring diversity of self-compassion and collectivistic vs. individualistic patterns on an individual level. Notwithstanding these limitations, our study reinforces literature that has shown that both self-compassion and self-coldness mediate the relationship between perceived social support, on the one hand, and wellbeing and psychological distress, on the other (e.g., Wilson et al., 2020). More importantly, while the direct association between perceived social support and wellbeing facets was not moderated by culture, a moderating effect of culture was found for the relationship mediated by self-compassion. This revealed cultural moderation reinforces the importance of considering cultural context when designing support interventions aimed at promoting wellbeing by enhancing self-compassion. Given that the indirect effect through self-compassion is culturally moderated, mental health strategies that aim to improve perceived social support and self-compassion should be culturally sensitive. For example, interventions might need to be adjusted to better suit the cultural values and norms of different groups to maximize their effectiveness.

The current findings add to growing research supporting the significant role that self-compassion and self-coldness can play in unraveling the relationship between perceived social support and wellbeing (Chan et al., 2023; Wilson et al., 2020). It also sheds light on the diverse behavior of self-compassion and self-coldness in different cultural contexts, supporting the perception that self-compassion is better studied as composed of two constructs. Investigating these nuanced cultural factors could illuminate the specific mechanisms through which cultural context moderates the mediation pathways between social support and mental health. Such exploration would provide deeper insights into why and how these relationships manifest differently across cultural groups, potentially informing more culturally sensitive interventions.

## Data Availability

The raw data supporting the conclusions of this article will be made available by the authors, without undue reservation.
